# IMPACT OF KRAS MUTATIONS IN CLINICAL FEATURES IN COLORECTAL CANCER

**DOI:** 10.1590/0102-672020200003e1524

**Published:** 2020-12-16

**Authors:** Renato Morato ZANATTO, Gianni SANTOS, Júnea Caris OLIVEIRA, Eduardo Marcucci PRACUCHO, Adauto José Ferreira NUNES, Gaspar Jesus LOPES-FILHO, Sarhan Sydney SAAD

**Affiliations:** 1 Department of Abdominal and Pelvic Surgery, Hospital Amaral Carvalho, Jaú, SP, Brazil; 2 Department of Biostatistics, Federal University of São Paulo, São Paulo, SP, Brazil; 3 Pathology Department, Hospital Amaral Carvalho, Jaú, SP, Brazil; 4 Department of Digestive Surgery, Federal University of São Paulo, São Paulo, SP, Brazil; 5 Postgraduate Program in Surgery, Federal University of São Paulo, São Paulo, SP, Brazil

**Keywords:** Colorectal neoplasms, Gene frequency, Mutation, Neoplasias colorretais, Frequência do gene, Mutação

## Abstract

**Background:**

KRAS mutations are important events in colorectal carcinogenesis, as well as negative predictors of response to EGFR inhibitors treatment.

**Aim:**

To investigate the association of clinical-pathological features with KRAS mutations in colorectal cancer patients treated.

**Methods:**

Data from 69 patients with colorectal cancer either metastatic at diagnosis or later, were retrospectively analyzed. The direct sequencing and pyrosequencing techniques were related to KRAS exon 2. The mutation diagnosis and its type were determined.

**Results:**

KRAS mutation was identified in 43.4% of patients. The most common was c.35G>T (p.G12V), c.35G>A (p.G12D) and c.38G>A (p.G13D). No correlation was found between KRAS mutation and age (p=0.646) or gender (p=0.815). However, mutated group had higher CEA levels at admission (p=0.048) and codon 13 mutation was associated with involvement of more than one metastatic site in disease progression (p=0.029). Although there was no association between primary tumor site and mutation diagnosis (p=0.568), primary colon was associated with worse overall survival (p=0.009).

**Conclusion:**

The KRAS mutation was identified in almost half of patients. Mutated KRAS group had higher levels of CEA at admission and the mutation at codon 13 was associated with involvement of more than one metastatic site in the course of the disease. Colon disease was associated with the worst overall survival.

## RESUMO

**Racional:**

Mutações KRAS são eventos importantes na carcinogênese colorretal como preditores negativos de resposta ao tratamento.

**Objetivo:**

Investigar a associação de características clinicopatológicas com mutações no KRAS em pacientes com câncer colorretal tratados.

**Métodos:**

Sessenta e nove pacientes com câncer colorretal metastáticos ao diagnóstico ou posteriormente foram analisados. As técnicas de sequenciamento direto e pirosequenciamento foram relacionadas ao éxon 2 do KRAS e o diagnóstico da mutação e seu tipo foram determinados.

**Resultados:**

A mutação KRAS foi identificada em 43,4% dos pacientes, c.35G>T (p.G12V), c.35G>A (p.G12D) e c.38G>A (p.G13D). Não foi encontrada correlação entre a mutação KRAS e a idade (p=0,646) ou o gênero (p=0,815). No entanto, o grupo mutado apresentou níveis mais altos de CEA na admissão (p=0,048). A mutação do códon 13 foi associada ao envolvimento de mais de um local metastático na progressão da doença (p=0,029); não houve associação entre o local primário do tumor e o diagnóstico de mutação (p=0,568); a doença primária do cólon foi associada com pior sobrevida global (p=0,009).

**Conclusão:**

A mutação KRAS foi identificada em quase metade dos pacientes. O grupo KRAS mutado apresentou níveis mais altos de CEA na admissão e a mutação no códon 13 foi associada ao envolvimento de mais de um local metastático no curso da doença. A doença do cólon foi associada com pior sobrevida global.

## INTRODUCTION

Colorectal cancer (CRC) is a common and lethal disease. It is the third most frequently diagnosed cancer in men and the second in women worldwide, with 1.65 million new cases and almost 835,000 deaths in 20157. In Brazil, for the 2018-2019 biennium, an estimated 36,360 new cases occur every year^14^.

The treatment is based on the presentation of the disease, that is, depending on the location and stage of the tumor, strategies that use surgical treatment, combined or not with chemotherapy and radiotherapy. Most patients are not expected to be cured in situations of metastatic disease, except for those with isolated liver and/or pulmonary involvement that can be treated with curative intent and rescued with operations. Most patients are treated with systemic palliative chemotherapy, with the clinical objective of improving quality of life and survival^21^.

Recently, epidermal growth factor receptor (EGFR) inhibitors, such as cetuximab and panitumumab, have been incorporated into the treatment of metastatic CRC in combination with chemotherapy. They act in blocking receptors and have demonstrated improved treatment efficacy for many tumors. In metastatic colorectal cancer, it resulted in a progression-free survival gain and a significant benefit has been reported in the continuation of these drugs after progression as a first-line treatment^27^ ^,^ ^5^. However, EGFR inhibitors are ineffective when KRAS is mutated^26^. The mutation rate varies from 30-50% and causes continuous activation of the EGFR intracellular pathway, regardless of the pharmacological blockade of the receptor, promoting tumor proliferation and survival^23^ ^,^ ^13^. Therefore, since the KRAS mutation is a predictor of a negative response, mutational analysis of the gene becomes mandatory before the institution of treatment with EGFR inhibitors. This strategy, in addition to optimizing health costs, avoids adverse effects related to these drugs, especially skin toxicity^19^ ^,^ ^16^.

Approximately 90% of the genetic mutations in the RAS family (H, N and K-RAS) occur in exon 2 of KRAS (codons 12 and 13). The most frequent mutation of codon 12 is c.35G> A (p.G12D) and at codon 13 is c.38G> A (p.G13D), both result from the exchange of the amino acid glycine for aspartic acid in positions 35 and 38, respectively^19^. Other mutations in KRAS such as exon 3 (codons 59, 60, 61), exon 4 (codons 119, 146, 147) and NRAS represent a small proportion of these mutations^11^.

The KRAS mutation is a predictor of a negative response to treatment with EGFR inhibitors and, according to other studies, confers a worse prognosis, but not in all patients^3^ ^,^ ^24^ ^,^ ^6^.

Therefore, this study aims to analyze the frequency, the types of mutation of the KRAS gene and the correlation with clinical and pathological data in patients diagnosed with metastatic CRC.

## METHODS

### Study design and ethical standards

Retrospective, transversal and single center study. All clinical information was obtained from medical records and included patients diagnosed and treated between August 2005 and February 2017. The study was reviewed and approved by the Research Ethics Committee of Hospital Amaral Carvalho on November 3, 2016 under the registration number 1,803,348. Individual consent for patient participation was not required due to the retrospective nature of the study and is in accordance with Brazilian regulatory legislation.

Sixty-nine patients with colon and rectal adenocarcinoma were evaluated, regardless of the treatment performed. They were divided into two groups: metastatic at diagnosis (n=43) and who developed metastases during postoperative oncological follow-up (n=26). The clinicopathological characteristics recorded and analyzed included age, gender, primary tumor site, metastasis and CEA levels at admission. All paraffin blocks were tested in representative areas of the tumor and sent to the laboratories to perform the extraction and sequencing of KRAS exon 2 DNA (codons 12 and 13). The DNA samples were derived from a primary tumor (n=58) or metastasis (n=11). Twenty-four were sequenced at Progenetics Molecular Diagnostics using the direct sequencing technique and 45 samples at the AC Camargo Cancer Center using the pyrosequencing technique^13^ ^,^ ^10^.

### Statistical analysis

After descriptive analysis of the data, two inferential analyzes were performed to confirm or refute the correlation: Pearson’s chi-square test or Fisher’s exact extension test, comparing gender, age, primary location, CEA level at admission, pathological staging and metastatic location according to the mutation of the KRAS1 gene, Cox multiple regression comparing the survival time, primary site and mutation of the KRAS12 gene. In all conclusions obtained through inferential analyzes, the level of alpha significance was 5%. The data was inserted and stored in Excel 2010 for Windows spreadsheets. Statistical analyzes were performed using the statistical program R, version 3.3.225.

## RESULTS

**FIGURE 1:**
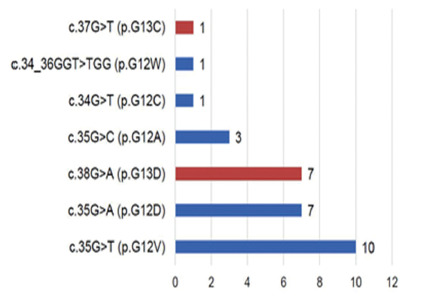
Types of mutations and corresponding amino acid changes

The KRAS mutation was diagnosed in 30 patients (43.5%). Twenty-two were at codon 12 (73.3%) and eight (26.7%) at codon 13. The most frequent mutations were c.35G> T (p.G12V), 33.3%, followed by mutation c .35G> A (p.G12D), 23.3% and c.38G> A (p.G13D), 23, 3%. These three mutations corresponded to 79.9% of the total mutations in the series. In one case, the double mutation c.34_36GGT> TGG (p.G12W), 0.03%, was identified, which involves the exchange of the amino acid glycine for tyrosine at position 34 and tyrosine for glycine at position 36 (Figure 1).

The correlation of the KRAS mutation with epidemiological data is described in Table 1. The wild KRAS group was composed mainly of men (53.8%), over 50 years old (71.8%), with primary colon location (66,7%), CEA admission level of up to 5 ng/ml (53.8%) and pathological staging IV (64.1%). The preferred metastatic site was the liver (46.2%), followed by peritoneum (12.8%), retroperitoneal lymph node (7.7%) and lung (5.1%). The KRAS mutated group was also formed by the majority with men (56.7%), over 50 years old (66.7%), primary colon site (60.0%), CEA level at admission above 5 ng/ml (70.0%) and pathological staging IV (60.0%). The preferred metastatic site was liver (43.3%), and after lung (23.3%), peritoneum (10.0%) and retroperitoneal lymph node (3.3%). The KRAS mutation was not related to gender (p=0.815), age group (p=0.646), primary site (p=0.568), pathological staging (p=0.935) and metastatic site (p=0.263). Patients with mutated KRAS had higher levels of CEA when compared to those with tumors with wild-type KRAS. Mutated KRAS patients had CEA levels greater than 5 ng/ml in 70% of cases, against 46.2% of those with wild KRAS (p=0.048).

**TABLE 1:** Distribution of the general characteristics of the patients according to the KRAS mutation

	KRAS Wild	%	KRAS Mutated	%	Total	%	p
Gender							0.815a
Men	21	(53.8%)	17	(56.7%)	38	(55.1%)	
Women	18	(46.2%)	13	(43.3%)	31	(44.9%)	
Total	39	(100%)	30	(100%)	69	(100%)	
Age range							0.646a
Up to 50 y	11	(28.2%)	10	(33.3%)	21	(30.4%)	
Above 50 y	28	(71.8%)	20	(66.7%)	48	(69.6%)	
Total	39	(100%)	30	(100%)	69	(100.0%)	
Primaru site							0.568a
Colon	26	(66.7%)	18	(60.0%)	44	(63.8%)	
Rectum	13	(33.3%)	12	(40%)	25	(36.2%)	
Total	39	(100%)	30	(100%)	69	(100%)	
CEA level at admission							
Up to 5 ng/ml	21	(53.8%)	9	(30.0%)	30	(43.5%)	0.048a
Above 5 ng/ml	18	(46.2%)	21	(70.0%)	39	(56.5%)	
Total	39	(100%)	30	(100%)	69	(100%)	
Pathological stage							
I	1	(2.6%)	-	-	1	(1.4%)	0.935b
II	7	(17.9%)	6	(20.0%)	13	(18.8%)	
III	6	(15.4%)	6	(20.0%)	12	(17.4%)	
IV	25	(64.1%)	18	(60.0%)	43	(62.3%)	
Total	39	(100%)	30	(100%)	69	(100%)	
Metastatic site							
Liver	18	(46.2%)	13	(43.3%)	31	(44.9%)	0.263b
Peritoneum	5	(12.8%)	3	(10.0%)	8	(11.6%)	
Lung	2	(5.1%)	7	(23.3%)	9	(13.0%)	
Retroperitoneum lymph node	3	(7.7%)	1	(3.3%)	4	(5.8%)	
More than a site	11	(28.2%)	6	(20.0%)	17	(24.6%)	
Total	39	(100%)	30	(100%)	69	(100%)	

The distribution of the number of metastatic sites, according to the type of mutation in the CRC, is described in Table 2. Of the 30 patients with KRAS mutation, a mutation was found in codon 12 (22), most had only one metastatic site (90.9%). The same did not occur with patients with mutations in codon 13 (8), of which half (50.0%) had only one metastatic site (p=0.029).

**TABLE 2:** Distribution of the number of metastasis sites, according to the type of KRAS mutation

	Number of metastatic sites					
Mutation type	Only one		More than one		Total	
	n	%	n	%	n	%
p.G12A	3	12.5%	-	-	3	10.0%
p.G12C	1	4.2%	-	-	1	3.3%
p.G12D	7	29.2%	-	-	7	23.3%
p.G12V	8	33.3%	2	33.3%	10	33.3%
p.G13C	-	-	1	16.7%	1	3.3%
p.G13D	4	16.7%	3	50.0%	7	23.3%
p.G12W	1	4.2%	-	-	1	3.3%
Total	24	100%	6	100%	30	100%

n=mutation number

**FIGURE 2:**
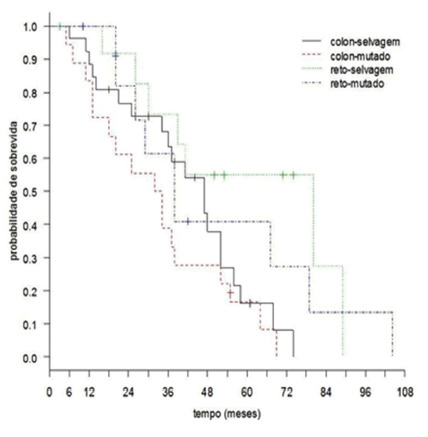
Patients’ overall survival curve, according to primary site and KRAS mutation

Figure 2 illustrates the patient’s overall survival by primary site and KRAS status. According to it, the overall survival of patients estimated at 60 months was 26.7%. Investigating survival, according to the primary site and KRAS, Cox’s regression model was adjusted, and the effect of the interaction between the primary site and KRAS status was not detected (p=0.961). Thus, the primary survival site was not influenced by KRAS. The results revealed that patients with primary rectal tumors had better survival when compared to those diagnosed with primary colon tumors (p=0.009). It was not possible to show a KRAS-type relationship with patient survival (p=0.144).

## DISCUSSION

In this study, the prevalence of the KRAS mutation was 43.4%. In most series, it is identified in 30-50% of cases and are important genetic factors that contribute to the occurrence of the disease^13^. The most frequent mutations were c.35G> T (p.G12V), 33.3%, c.35G> A (p.G12D), 23.3% and c.38G> A (p.G13D), 23, 3% that corresponded to 79.9% of the total mutations in this sample. Reports show a 39.3% prevalence of mutations in KRAS among 1018 German patients, and the most prevalent mutation was p.G12D followed by p.G12V and p.G13D19.

In Brazil, variations in the prevalence of the KRAS mutation are observed according to the region, for example, about 18.3% among 60 patients in the Amazon region^9^. In the largest Brazilian study that evaluated 8234 patients, the frequency of this mutation was 31.9%. In the analysis of separate regions, in the southeastern region it was 34.7%^13^.

Therefore, the codon distribution and the three most prevalent mutations in the present study are similar to those described in other series, denoting a representative sample compared to the global findings^13^ ^,^ ^29^ ^,^ ^28^.

In the present study, the frequency of KRAS mutation did not correlate with gender (p=0.815), age group (p=0.646), primary site (p=0.568), pathological staging (p=0.935) and metastatic site (p=0.263). Reports show a high prevalence among women (34.8% women vs. 32.5% men, p=0.03)^13^. Thus, gender, age or hormonal influence on the KRAS mutation are conflicting because some studies show a higher frequency of mutations for women^22^, but others do not^17^. The ethnological differences in the populations studied may explain the disparity.

Regarding the primary site and the multimodal therapy generally used, two groups of patients were identified: extraperitoneal rectum, in which radiotherapy is part of the local therapy, plus surgical resection and adjuvant chemotherapy, and intraperitoneal colon/rectum, in which the resection operation is the basis of local therapy, with complementary adjuvant chemotherapy according to risk stratification. In addition, systemic chemotherapy with fluoropyrimidine-based chemotherapy, with or without panitumumab/cetuximab or bevacizumab monoclonal antibody is the basis of therapy for patients with metastatic disease. Although the KRAS mutation is not correlated with the primary site in the present study, by a different group, tumors on the right side (before the left flexure) and tumors on the left side (after the left flexure) showed influence of the primary site on the prognosis in previously untreated tumors. However, these data are still the subject of investigation^18^.

The mutation status of KRAS appears to influence the pattern of metastatic spread in CRC. Studies show a difference in the frequency of KRAS mutations in patients with liver, lung and brain metastases^24^. In the study, KRAS mutations were less prevalent in liver metastases (32.3%), but more in pulmonary (62.0%) and brain (56.5%, p=0.003). There are some reports that metastatic CRC with KRAS mutation is more likely to spread to the lungs compared to wild-type KRAS (22% vs. 13%, p<0.01)^30^. Although this information was not reported in the present study, mutations in the codon distribution were correlated with the number of affected metastatic sites and, in codon 12, most had only one metastatic site. The same did not happen with the codon 13 mutation group, because half of the group had more than one metastatic site (p=0.029).

In the mutated KRAS group, CEA levels above 5 ng/ml were identified more frequently when compared to the wild-type KRAS group (p=0.048). A recent study does not show a correlation between CEA levels (cut-off point 200) and KRAS status among 193 patients with metastatic CRC^8^. Therefore, it is difficult to sustain the correlation between CEA levels and KRAS status due to the heterogeneity of the cutoff points, the diversified laboratory methodology employed, the number and ethnicity of patients between studies.

The overall estimated 60-month survival rate for patients was 26.7%. The effect of the interaction between the primary site and the KRAS status was not detected (p=0.961). In addition, the results revealed that patients with rectal location had longer survival when compared to those with location in the colon (p=0.009). It was not possible to show a KRAS-type relationship with survival (p=0.144).

The KRAS mutation provides a worse prognosis, as shown in many studies^3^ ^,^ ^24^, but some discordant findings have been reported^6^. One explanation for this controversy is that different mutations in the same gene can cause different prognostic influences. For example, in the multicenter RASCAL study, the prognostic significance of mutations at codon 12 or 13 in 2721 patients from 13 countries was assessed. Multivariate analysis showed that only codon 12 mutations were independently associated with an increased risk of recurrence and death. However, after expanding the sample to include 4268 patients, subsequent analyzes show 12 possible mutations in codons 12 and 13, but only one specific mutation in codon 12 (9%) was significantly associated with an adverse outcome, and especially in patients with lymph nodes positive^2^. Although less information is available, mutations in the NRAS are also associated with a worse prognosis^4^

Although the effectiveness of the treatment and the evaluation of clinical results are not the objectives of this study, it can also influence survival, since the results of the frequency of RAS mutations can also, but unfortunately these data were not available (except KRAS exon 2). In addition, considering the possible impact of RAS mutations on the overall results of the analyzed population, this may not be significant due to the few frequencies of non-KRAS exon 2 mutations recorded. In addition, it was only in 2015 that the American Society of Clinical Oncology (ASCO) recommended the prolonged use of the RAS test for all patients who are candidates for treatment with EGRF inhibitors.

The DNA samples in this study were derived from a primary tumor or metastasis, because the literature showed that mutations in KRAS, NRAS and BRAF are similar in both types of samples^15,20,29^ and there is no disagreement in the status of KRAS in different periods of patients evaluated, including at the time of the initial diagnosis of the tumor and later in the course of the disease in the metastatic period and after cytotoxic chemotherapy. However, challenging data have recently been demonstrated that the primary tumor test at a single site in synchronic metastatic colorectal disease can result in an incomplete profile of the KRAS mutation and, consequently, an incorrect choice of the use of EGFR inhibitors as treatment^10^. This study showed a statistically significant difference in survival in Brazilian metastatic patients with primary colon tumors treated at a medical oncology center, according to the established routine of clinical practice. There is an expectation that, with future research, it will lead to expanding our recognition by characterizing several biomarkers involved in colorectal carcinogenesis and establishing clinicopathological variables to seek new effective and personalized treatments.

## CONCLUSION

The KRAS mutation was identified in almost half of the patients. Elevated levels of CEA were seen more frequently in the mutated group, and the codon 13 mutation was associated with the involvement of more than one metastatic site in the course of the disease. Primary colon disease was associated with worse overall survival. None of the other clinicopathological characteristics evaluated were related to mutation presence.
